# Equity in human papilloma virus vaccination uptake?: sexual behaviour, knowledge and demographics in a cross-sectional study in (un)vaccinated girls in the Netherlands

**DOI:** 10.1186/1471-2458-14-288

**Published:** 2014-03-28

**Authors:** Madelief Mollers, Karin Lubbers, Symen K Spoelstra, Willibrord CM Weijmar-Schultz, Toos Daemen, Tjalke A Westra, Marianne AB van der Sande, Hans W Nijman, Hester E de Melker, Adriana Tami

**Affiliations:** 1 Epidemiology and Surveillance, Centre for Infectious Disease Control, National Institute for Public Health and the Environment (RIVM), Bilthoven, The Netherlands; 2 Pathology, VU University Medical Center (VUmc), Amsterdam, The Netherlands; 3 Department of Obstetrics and Gynecology, University of Groningen, University Medical Center Groningen, Groningen, The Netherlands; 4 Department of Medical Microbiology, Molecular Virology Section, University of Groningen, University Medical Center Groningen, Groningen, The Netherlands; 5 Julius Center for Health Sciences and Primary Care, University Medical Center Utrecht, Utrecht, The Netherlands; 6 Department of Medical Microbiology, University Medical Center Groningen, P.O. Box 30.001, 9700 RB Groningen, The Netherlands

**Keywords:** Monitoring, Human papilloma virus, Vaccination uptake, Sexual behaviour, Knowledge

## Abstract

**Background:**

In the Netherlands, human papillomavirus (HPV) vaccination is part of a national program equally accessible for all girls invited for vaccination. To assess possible inequalities in vaccine uptake, we investigated differences between vaccinated and unvaccinated girls with regard to various characteristics, including education and ethnicity, (both associated with non-attendance to the national cervical screening program), sexual behaviour and knowledge of HPV.

**Methods:**

In 2010, 19,939 nationwide randomly-selected 16–17 year-old girls (2009 vaccination campaign) were invited to fill out an online questionnaire. A knowledge scale score and multivariable analyses identified variables associated with vaccination status.

**Results:**

2989 (15%) of the selected girls participated (65% vaccinated, 35% unvaccinated). The participants were comparable with regard to education, ethnicity, most sexual risk behaviour and had similar knowledge scores on HPV transmission and vaccination. However, unvaccinated girls lived in more urbanised areas and were more likely to have a religious background. Irrespective of vaccination status, 81% of the girls were aware of the causal relationship between HPV and cervical cancer, but the awareness of the necessity of cervical screening despite being vaccinated was limited.

**Conclusions:**

HPV vaccine uptake was not associated with knowledge of HPV and with factors that are known to be associated with non-attendance to the cervical cancer screening program in the Netherlands. Furthermore, most sexual behaviour was not related to vaccination status meaning that teenage unvaccinated girls were not at a disproportionally higher risk of being exposed to HPV. Routine HPV vaccination may reduce the social inequity of prevention of cervical cancer.

## Background

Human papillomavirus (HPV) infection is one of the most common sexually transmitted infections (STI) worldwide. By the age of 50, about 80% of sexually active women will acquire HPV [1-3]. Most infections are transient and around 90% clears within two years [4,5]. However, a persisting high-risk HPV infection is the most important risk factor for the development of premalignant cervical intraepithelial neoplasia (CIN1-3) and cervical cancer [6].

Since 2006, a quadrivalent vaccine that induces protection against HPV types 6, 11, 16 and 18 (Gardasil®), and in 2007 a bivalent vaccine against HPV type 16 and 18 (Cervarix®) are available. Both have shown an efficacy of >90% in preventing CIN2/3 [7,8].

In the Netherlands, the bivalent HPV vaccine (Cervarix®) targeting 12-year-old girls became part of the National Immunisation Program in 2010. In 2009, girls aged 13 to 16 years were offered this vaccine during a “catch-up” vaccination program where the uptake amounted to 52% [9]. HPV vaccination uptake depends on diverse factors. In the United Kingdom, schoolgirls from ethnic minorities had a lower vaccine uptake and in the United States, girls with lower socio-economic status were less likely to take up the full 3 doses of the vaccine [10,11]. In the Netherlands, a cervical cancer screening program runs since 1976. Currently, the program provides organized cervical cytological screening every five years for women aged 30–60 years. In 2005, there was a 65% attendance to the cervical screening program [12]. However, when including opportunistic screening figures, the overall coverage for cervical screening amounted to 77% nationwide [13]. It has been shown that in the Netherlands, socio-economically disadvantaged women and women of non–Dutch nationality attend the national cervical screening program less frequently [14]. More than half of the cervical cancers are diagnosed in women who do not attend the Dutch screening program [15,16]. Reducing the occurrence of cervical cancer might thus be hampered if girls who tend to decline HPV vaccination now are also screened less often in the future. This might lead to inequity, i.e. unequal fairness or justice in the way people are treated.

Prior to introduction of vaccination, some studies have reported on girls’ intention to be vaccinated [17-19]. However, little is known about the characteristics of girls in relation to actual HPV vaccination uptake [20,21]. This study aims to explore differences between vaccinated and unvaccinated girls with regard to characteristics such as education, ethnicity, (sexual) risk behaviour and knowledge of HPV. Understanding the features of these two groups could provide insight in future vaccine and screening targeting efforts.

## Methods

### Study population and study design

A nationwide self-reported cross-sectional study of 16-17-year-old girls was performed in the Netherlands in December 2010. A random sample of 19,939 girls born in 1993, invited for the HPV catch-up vaccination campaign in 2009 (9992 vaccinated and 9947 unvaccinated), was selected from the national vaccination database (Praeventis) held at the National Institute for Public Health and the Environment (RIVM) [22].

A semi-structured questionnaire was developed, pre-tested and applied to the study population via online research survey software (EFS Survey version 8.1, Unipark (Questback)). The questionnaire contained pre-coded questions on the following topics: *Socio-demographic*: education level (girl and parents), ethnicity (Dutch, Turkish, Moroccans, Surinamese, Antilleans, Arubans and other), religion, alcohol and smoking behaviour. *Sexual behaviour*: e.g. ever have had sexual contact (vaginal or anal), age of sexual debut, use of contraceptives (especially condom use), number of sexual partners, type of current partner (steady/casual) and history of STI. *HPV knowledge and HPV vaccination:* e.g. modes of transmission, protection level of HPV vaccination, risk of infection and participation in the cervical cancer screening program. Each girl selected for the study received an invitation by post with an information letter for the girl and one for her parents/caretakers, and a link and a unique code to access the online questionnaire. The questionnaire took approximately ten minutes to fill out and the information was processed anonymously.

This research was performed according to the principles contained in the Declaration of Helsinki [23]. The Dutch Central Committee on Research Involving Human Subjects (Centrale Commissie Mensgebonden Onderzoek (CCMO)) decided that the nature of the study did not require mandatory approval of a competent medical-ethical review committee, in agreement with the Dutch Medical Research involving Human Subjects Act. The CCMO allowed to receive consent from the participating girls through the online system (no written consent from the girls or their parents was required).

### Statistical analysis

Differences in socio-demographic characteristics, sexual behaviour and knowledge of HPV between vaccinated and unvaccinated women were compared in contingency tables using χ^2^ test. Socio-economic status was also assessed using the ‘status score’ computed by the Dutch Institute for Social Research (SCP, http://www.scp.nl), as a proxy for socioeconomic status (SES). This score takes into account the average income per household in a given postcode area as well as the percentage of households with low income, without a paid job and with low education level. The lower the score is, the higher the socioeconomic status [24]. Knowledge scale composite scores were calculated assigning a point for each correct answer to 8 general knowledge questions (0–8) (general knowledge score) and to 10 questions on HPV transmission knowledge (0–10) (transmission knowledge score). Mean scores were compared for vaccinated and unvaccinated girls by a t-test. Significance was determined at the 5% level (P-value ≤0.05).

Variables associated with vaccination status found to approach significance (P-value ≤0.1) were fitted in a multivariable logistic regression model. The strength of the associations was expressed as crude odds ratios (OR) in univariable analysis and as adjusted OR (aOR) in multivariable analysis, comparing vaccinated vs. unvaccinated girls. Two models were carried out: one including the total sample of girls and the second comprising only sexually active girls. The final models included those factors that remained significant (P-value ≤0.05) after backward selection and those found to change the OR of other variables by at least 10%. Analyses were conducted using software packages from SAS 9.2 (SAS Institute Inc. 2010, USA).

## Results

### Socio-demographic and sexual behaviour characteristics

A total of 2989 females (15% of invited participants) aged 16–17 (median 17) participated in this study. Among them, 65% received at least 1 HPV vaccination and 35% received none. The distribution of most characteristics was similar between vaccinated and unvaccinated girls (Tables 1 and 2). However, vaccinated girls were more likely to live in low urbanised areas and less likely had a religious background. Amongst those who professed a religion, vaccinated girls were more often Catholic while unvaccinated girls were more often Protestant Christian. The vaccination status between the different non-Dutch ethnic subgroups was similar (P = 0.833), they were therefore grouped together into a “non-Dutch” ethnic group for analysis. Ethnicity was not associated with vaccination status (Table 1). Drinking alcohol was reported more by vaccinated girls as was the use of contraceptives (Table 1). A slightly higher percentage of vaccinated girls were sexually active but amongst them a lower mean total number of sexual partners in their lifetime was identified. Except for the higher percentage of female partners in the unvaccinated girls, there were no other differences in sexual behaviour (Table 2).

**Table 1:** Socio-demographic characteristics amongst all girls participating in our study (n = 2989), the Netherlands 2010

**Category**	**Total**	**Vaccinated**	**Unvaccinated**	**OR (95% CI) for being vaccinated**	**P-value**
	**N (%)**	**n (%)**	**n (%)**		
	2989 (100)	1938 (65)	1051 (35)		
**Age**				**ref**	
Median (range)	17 (16–17)	17 (16–17)	17 (16–17)	0.9 (0.6–1.2)	0.45
**Ethnicity** (n=2988)					
Dutch	2849 (95)	1852 (96)	997 (95)	**ref**	
Non–Dutch	139 (5)	86 (4)	53 (5)	0.9 (0.6–1.2)	0.45
**Degree of urbanisation** (n=2971)					
Low (1–1000 inhabitants)	1514 (51)	1019 (53)	495 (47)	**ref**	
High (>1000 inhabitants)	1457 (49)	908 (47)	549 (53)	0.8 (0.7–0.9)	0.004
**Socio economic status**^**a**^ (n=2962)					
Mean (95% CI)	−0.01(−0.03–0.03)	−0.02 (−0.06–0.02)	0.02 (−0.03–0.07)	0.9 (0.9–1.0)	0.20
**Religion** (n=2898)					
No religion	1532 (53)	1050 (56)	482 (48)	**ref**	
Catholics	708 (24)	511 (27)	197 (19)	1.2 (1.0–1.5)	
Protestant Christian	642 (22)	317 (17)	325 (32)	0.4 (0.4–0.5)	
Other	16 (1)	6 (0.3)	10 (1)	0.3 (0.1–0.7)	<0.001
**Education**^**b**^					
Low	221 (7)	148 (8)	73 (7)	**ref**	
Middle	992 (33)	631 (33)	361 (34)	0.9 (0.6–1.2)	
High	1776 (59)	1159 (60)	617 (59)	0.9 (0.7–1.2)	0.54
**Education of parents**^**b**^ (n=2787)					
Low	49 (2)	28 (2)	21(2)	**ref**	
Middle	1162 (42)	749 (41)	413 (42)	1.4 (0.8–2.4)	
High	1576 (57)	1031 (57)	545 (56)	1.4 (0.8–2.5)	0.45
**Alcohol use** (n=2983)					
No	723 (24)	425 (22)	298 (28)	**ref**	
Yes	2260 (76)	1509 (78)	751 (72)	1.4 (1.2–1.7)	<0.001
**Smoking** (n=2984)					
No	2280 (76)	1492 (77)	788 (75)	**ref**	
Current smoker	476 (16)	299 (15)	177 (17)	0.9 (0.7–1.2)	
Former smoker	228 (8)	144 (7)	84 (8)	0.9 (0.7–1.1)	0.47
**Contraception** (n=2910)					
No	902 (31)	533 (28)	369 (37)	**ref**	
Yes	2008 (69)	1371 (72)	637 (63)	1.5 (1.3–1.8)	<0.001
**Type of contraception** (n=2003)					
Pill	1253 (63)	848 (62)	405 (64)	**ref**	
Condom	160 (8)	100 (7)	60 (9)	0.8 (0.6–1.1)	
Pill and condom	544 (27)	385 (28)	159 (25)	1.2 (0.9–1.4)	
Other	46 (2)	34 (2)	12 (2)	1.4 (0.7–2.7)	0.18
**Ever had sex** (n=2898)					
No	1303 (45)	826 (44)	477 (48)	**ref**	
Yes	1595 (55)	1070 (56)	525 (52)	1.2 (1.0–1.4)	0.04

^a^Combination of the average income per household with percentage of households with low income, without a paid job and with low average education resulting in a score ranging [−4;+4]. Note that the lower the score is, the higher the socioeconomic status is [24].

^b^Low = no education or primary education; Middle = junior technical school, lower general or intermediate vocational secondary education; High = higher vocational or higher general secondary education, pre-university/university education.

Missing values are deducted from the total number of girls.

**Table 2:** Sexual risk factors amongst sexually active girls participating in our study (n = 1595), the Netherlands 2010

**Category**	**Total**	**Vaccinated**	**Unvaccinated**	**OR (95% CI) for being vaccinated**	**P-value**
	**N (%)**	**n (%)**	**n (%)**		
	1595 (100)	1070 (67)	525 (33)		
**Steady partner** (n=1588)					
No	510 (32)	336 (32)	174 (33)	**ref**	
Yes	1078 (68)	729 (68)	349 (67)	1.1 (0.9–1.4)	0.49
**Number of casual partners** (n=1589)					
0	806 (51)	553 (52)	253 (48)	**ref**	
1	602 (38)	402 (38)	200 (38)	0.9 (0.7–1.2)	
2	125 (8)	75 (7)	50 (10)	0.7 (0.5–1.0)	
>2	56 (4)	35 (3)	21 (4)	0.8 (0.4–1.4)	0.24
**Condom use steady partner** (n=1078)					
Always	193 (18)	126 (17)	67 (19)	**ref**	
Not always	885 (82)	603 (83)	282 (81)	1.1 (0.8–1.6)	0.44
**Condom use casual partner** (n=778)					
Always	219 (28)	144 (28)	75 (28)	**ref**	
Not always	559 (72)	364 (72)	195 (72)	1.0 (0.7–1.3)	0.87
**STI** (n=780)					
No	641 (82)	423 (83)	218 (80)	**ref**	
Yes	139 (18)	86 (17)	53 (20)	0.8 (0.6–1.2)	0.36
**Sex of partner** (n=1589)					
Male	1560 (98)	1051 (99)	509 (97)	**ref**	
Female	29 (2)	15 (1)	14 (3)	0.5 (0.2–1.1)	0.08
**Age of sexual debut** (n=1579)					
Mean (95% CI)	15.5 (15.4–15.6)	15.5 (15.5–15.6)	15.4 (15.3–15.5)	1.2 (1.0–1.3)	0.74
**Total number of lifetime sexual partners** (n=1584)					
Mean (95% CI)	2.0 (1.9–2.1)	1.9 (1.8–2.0)	2.2 (2.0–2.4)	0.9 (0.9–1.0)	<0.001

Missing values are deducted from the total number of sexually active girls.

### Sexual behaviour and views associated with HPV vaccination

The majority of both vaccinated and unvaccinated girls (97.3% vs. 98.2%) thought that HPV vaccination had not changed their sexual behaviour, 0.8% of the girls answered that use of condoms would not be needed to protect against STIs after HPV vaccination. However, 17% of the vaccinated and 26% of the unvaccinated replied that other girls would be inclined to use less condoms after vaccination. In addition, a higher proportion of vaccinated girls thought HPV could not be transmitted when using condoms.

### General knowledge of HPV and HPV vaccination

Vaccinated girls were less aware that HPV vaccination does not protect against all HPV types, but were more aware that HPV vaccination does not protect against all STIs (Table 3). In general, few girls knew that HPV may cause genital warts (20%) and that most HPV infections clear on their own (5%). Approximately three quarters (73%) were aware that unprotected sex entails a higher risk of acquiring an HPV infection. More than 80% recognised that an HPV infection is a risk for cervical cancer and about 68% of the girls knew that an HPV infection does not always lead to cervical cancer. Also more than 80% knew that cervical cancer does not always lead to death. Depending on the question, a variable number of girls (9% to 63%) answered ‘don’t know’*.* The general knowledge score (one point for every right answer) was similar between vaccinated and unvaccinated girls.

**Table 3:** HPV general knowledge amongst all girls participating in our study (n = 2989), the Netherlands 2010

**Category**	**Total**	**Vaccinated**	**Unvaccinated**	**OR (95% CI) for being vaccinated**	**P-value**
	**N (%)**	**n (%)**	**n (%)**		
	2989 (100)	1938 (65)	1051 (35)		
**HPV vaccination protects against all HPV types** (n=2910)					
No	1811 (62)	1110 (58)	701 (70)	**ref**	
Yes	373 (13)	291 (15)	82 (8)	2.2 (1.7–2.9)	
Don’t know	726 (25)	505 (27)	221 (22)	1.4 (1.2–1.7)	<0.001
**HPV vaccination protects against all STIs** (n=2909)					
No	2567 (88)	1702 (89)	865 (86)	**ref**	
Yes	52 (2)	33 (2)	19 (2)	0.9 (0.5–1.6)	
Don’t know	290 (10)	170 (9)	120 (12)	0.7 (0.6–0.9)	0.03
**An HPV infection always leads to cervical cancer** (n=2930)					
No	1984 (68)	1294 (68)	690 (68)	**ref**	
Yes	121 (4)	80 (4)	41 (4)	1.0 (0.7–1.5)	
Don’t know	825 (28)	541 (28)	284 (28)	1.0 (0.9–1.2)	0.97
**Cervical cancer is always fatal** (n=2930)					
No	2414 (82)	1579 (82)	835 (82)	**ref**	
Yes	141 (5)	98 (5)	43 (4)	1.2 (0.8–1.8)	
Don’t know	375 (13)	238 (12)	137 (14)	0.9 (0.7–1.2)	0.43
**If you have unprotected sex, you are at high risk of an HPV infection** (n=2930)					
No	311 (11)	193 (10)	118 (12)	**ref**	
Yes	2143 (73)	1411 (74)	732 (72)	1.2 (0.9–1.5)	
Don’t know	476 (16)	311 (16)	165 (16)	1.2 (0.9–1.6)	0.42
**An HPV infection is a risk for cervical cancer** (n=2930)					
No	215 (7)	144 (8)	71 (7)	**ref**	
Yes	2370 (81)	1555 (81)	815 (80)	0.9 (0.7–1.3)	
Don’t know	345 (12)	216 (11)	129 (13)	0.8 (0.6–1.2)	0.48
**An HPV infection can cause genital warts** (n=2929)					
No	537 (18)	354 (18)	183 (18)	**ref**	
Yes	582 (20)	389 (20)	193 (19)	1.0 (0.8–1.3)	
Don’t know	1810 (62)	1172 (61)	638 (63)	1.0 (0.8–1.2)	0.63
**An HPV infection usually disappears on its own** (n=2930)					
No	1969 (67)	1301 (68)	668 (66)	**ref**	
Yes	136 (5)	80 (4)	56 (6)	0.7 (0.5–1.0)	
Don’t know	825 (28)	534 (28)	291 (29)	0.9 (0.8–1.1)	0.21
**Awareness of CC screening program** (n=2910)					
No	1421 (49)	984(52)	437 (43)	**ref**	
Yes	1489 (51)	921 (48)	568 (57)	0.7 (0.6–0.8)	<0.001
**Participation of mother in CC screening program** (n=2910)					
No	670 (23)	410 (22)	260 (26)	**ref**	
Yes	1192 (41)	798 (42)	394 (39)	1.3 (1.1–1.6)	
Don’t know	1048 (36)	697 (37)	351 (35)	1.3 (1.0–1.5)	0.03
**Need to participate in the CC screening program after vaccination** (n=2910)					
No	174 (6)	115 (6)	59 (6)	**ref**	
Yes	2013 (69)	1346 (71)	667 (66)	1.0 (0.7–1.4)	
Don’t know	723 (25)	444 (23)	279 (28)	0.8 (0.6–1.2)	0.03
**Condoms are not needed anymore once vaccinated** (n=2909)					
No	2781 (96)	1842 (97)	939 (94)	**ref**	
Yes	22 (1)	15 (1)	7 (1)	1.1 (0.5–2.9)	
Don’t know	106 (3)	48 (3)	58 (6)	0.4 (0.3–0.6)	<0.001
**Girls will use less condoms once vaccinated** (n=2909)					
No	1526 (52)	1076 (56)	450 (45)	**ref**	
Yes	574 (20)	317 (17)	257 (26)	0.5 (0.4–0.6)	
Don’t know	809 (28)	512 (27)	297 (30)	0.7 (0.6–0.9)	<0.001
**General knowledge score**					
mean (95% CI)	5.52 (5.48–5.56)	5.51 (5.47–5.56)	5.53 (5.47–5.59)		0.62

CC = cervical cancer.

Missing values are deducted from the total number of girls.

### Cervical screening

Vaccinated girls reported more often that their mother participated in the cervical cancer screening program, and that it would still be necessary to participate in such a program after being vaccinated. However, they were less aware of the existence of this program (Table 3).

### Knowledge on HPV infectiousness

Most girls were familiar with the association between unsafe vaginal sex and transmission of HPV. This knowledge was lower for other forms of sex, such as unsafe anal or oral sex. A low proportion knew that HPV could be transmitted via skin-to-skin contact or by stroking their partner’s genitals (Table 4). The HPV transmission knowledge score also showed no difference in univariable analysis.

**Table 4:** HPV transmission knowledge amongst all girls participating in our study (n = 2989), the Netherlands 2010

**Category**	**Total**	**Vaccinated**	**Unvaccinated**	**OR (95% CI) for being vaccinated**	**P-value**
	**N (%)**	**n (%)**	**n (%)**		
	2989 (100)	1938 (65)	1051 (35)		
**HPV can be transmitted via;**					
*Holding hands (no) (n=2924)*					
No	2884 (99)	1882 (99)	1002 (99)	**ref**	
Yes	40 (1)	28 (1)	12 (1)	1.2 (0.6–2.5)	0.53
*Deep throat kissing (no) (n=2927)*					
No	2622 (90)	1711 (89)	911 (90)	**ref**	
Yes	305 (10)	202 (11)	103 (10)	1.0 (0.8–1.3)	0.74
*Skin to skin contact (yes) (n=2926)*					
No	2646 (90)	1727 (90)	919 (91)	**ref**	
Yes	280 (10)	185 (10)	95 (9)	1.0 (0.8–1.3)	0.79
*Stroking partner at genitals (yes) (n=2927)*					
No	2000 (68)	1326 (69)	674 (66)	**ref**	
Yes	927 (32)	587 (31)	340 (34)	0.9 (0.7–1.0)	0.12
*Public toilet (no) (n=2926)*					
No	2411 (82)	1575 (82)	836 (83)	**ref**	
Yes	515 (18)	338 (18)	177 (17)	1.0 (0.8–1.2)	0.89
*Unprotected oral sex (yes) (n=2928)*					
No	1078 (37)	695 (36)	383 (38)	**ref**	
Yes	1850 (63)	1219 (64)	631 (62)	1.1 (0.9–1.2)	0.44
*Unprotected vaginal sex (yes) (n=2929)*					
No	144 (5)	96 (5)	48 (5)	**ref**	
Yes	2785 (95)	1818 (95)	967 (95)	0.9 (0.7–1.3)	0.73
*Unprotected anal sex (yes) (n=2928)*					
No	865 (30)	568 (30)	297 (29)	**ref**	
Yes	2063 (70)	1345 (70)	718 (71)	1.0 (0.8–1.2)	0.81
*Sex with a condom (n=2927)*					
No	2533 (87)	1674 (88)	859 (85)	**ref**	
Yes	394 (12)	238 (12)	156 (15)	0.8 (0.6–1.0)	0.03
*Sharing a spoon or cup (no) (n=2925)*					
No	2739 (94)	1777 (93)	962 (95)	**ref**	
Yes	186 (6)	134 (7)	52 (5)	1.4 (1.0–2.0)	0.05
*Sneezing/coughing (no) (n=2924)*					
No	2753 (94)	1794 (94)	959 (95)	**ref**	
Yes	171 (6)	116 (6)	55 (5)	1.1 (0.8–1.6)	0.48
**Transmission knowledge score**					
Mean (95% CI)	7.24 (7.19–7.28)	7.24 (7.16–7.31)	7.24 (7.18–7.29)		0.99

Missing values are deducted from the total number of girls.

Correct answer is shown in brackets.

### Multivariable analysis of predictors for vaccine receipt

Variables whose association with vaccination status approached significance (P-value ≤0.1) were fitted in a multivariable logistic regression model (Table 5). The factors that remained independently associated with being vaccinated were living in low urbanised areas, consuming alcohol, not being sexually active, using contraceptive methods and not being aware of the cervical cancer screening program. Additionally, girls ascribing to no religion and those who professed a Catholic faith were more likely to be vaccinated than those that were either Protestant or who belonged to another religion. Vaccinated girls were also more likely to report that their mothers participated in the cervical cancer screening program or not knowing their mother’s participation status in this screening program.

**Table 5:** Multivariable analysis amongst all girls participating in our study (n = 2989), the Netherlands 2010

**Risk factor**	**Univariable**^**a**^	**Multivariable**^**b,c**^	**P-value**
	**OR (95% CI)**	**aOR (95% CI)**	
**Degree of urbanisation**			
Low (1–1000 inhabitants)	**ref**	**ref**	
High (>1000 inhabitants)	0.8 (0.7–0.9)	0.8 (0.7–1.0)	0.02
**Religion**			
No religion	**ref**	**ref**	
Catholics	1.2 (1.0–1.5)	1.2 (0.9–1.4)	0.15
Protestant Christian	0.4 (0.4–0.5)	0.5 (0.4–0.6)	<0.001
Other	0.3 (0.1–0.7)	0.3 (0.1–0.9)	0.03
**Alcohol use**			
No	**ref**	**ref**	
Yes	1.4 (1.2–1.7)	1.3 (1.0–1.6)	0.01
**Contraception**			
No	**ref**	**ref**	
Yes	1.5 (1.3–1.8)	1.5 (1.2–1.9)	<0.001
**Ever had sex**			
No	**ref**	**ref**	
Yes	1.2 (1.0–1.4)	0.8 (0.6–1.0)	0.04
**Awareness of CC screening program**			
No	**ref**	**ref**	
Yes	0.7 (0.6–0.8)	0.7 (0.6–0.8)	<0.001
**Participation of mother to CC screening program**			
No	**ref**	**ref**	
Yes	1.3 (1.1–1.6)	1.6 (1.2–2.0)	<0.001
Don’t know	1.3 (1.0–1.5)	1.3 (1.0–1.6)	0.03
**HPV vaccination protects against all HPV types**			
No	**ref**	**ref**	
Yes	2.2 (1.7–2.9)	2.4 (1.8–3.2)	<0.001
Don’t know	1.4 (1.2–1.7)	1.5 (1.2–1.8)	<0.001
**Girls will use less condoms once vaccinated**			
No	**ref**	**ref**	
Yes	0.5 (0.4–0.6)	0.5 (0.4–0.6)	<0.001
Don’t know	0.7 (0.6–0.9)	0.7 (0.5–0.8)	0.002
**Condom use is not needed anymore once vaccinated**			
No	**ref**	**ref**	
Yes	1.1 (0.5–2.9)	1.5 (0.5–5.6)	0.5
Don’t know	0.4 (0.3–0.6)	0.4 (0.2–0.6)	<0.001

CC = cervical cancer.

^a^196 cases missing.

^b^Only variables with a P–value less than 0.1 in the univariable analysis were entered.

^c^not significant in multivariable analysis, (P > 0.05) (not presented in Table): HPV can be transmitted via a spoon, HPV can be transmitted when using condoms.

Furthermore, considering perceptions of HPV, vaccinated girls thought more often that HPV vaccination protects against all HPV types and were less likely to think that girls would use condoms less frequently once vaccinated. Interestingly, more unvaccinated girls did not know that condoms were still needed after vaccination.

## Discussion

This population-based study is one of the first that examined actual determinants for individual vaccine uptake instead of willingness to be vaccinated. Vaccinated and unvaccinated girls were comparable with regard to education, education of parents, ethnicity, most sexual risk behaviour and had similar scores on knowledge of HPV infection and HPV transmission. They differed with respect to characteristics such as urbanisation degree, religion, contraceptive use, number of lifetime sexual partners and importantly, their opinions on the use of condoms after HPV vaccination and the protection of vaccination against all HPV types.

Studies in the Netherlands have shown that women who are non-Dutch nationals and have a lower socio-economic status are less likely to participate in the cervical cancer screening program [14,15]. These risk factors for non-attendance were not observed in this study for girls who chose not to be vaccinated. We found no relationship between uptake and ethnicity, nor education of the girl or their parents or SES score (both indicators of socio-economic status), which could suggest that the two programs might strengthen each other Another Dutch study also concluded that vaccination and screening (assessed by reported screening behaviour of the mother) complement each other to a large extent [25].

Religion has influenced vaccination decision-making process since the beginning of vaccination efforts [26]. Anti-vaccination proponents were most common in countries with a high proportion of Protestants [27]. In our study, identifying as a Protestant Christian was related to a lower vaccination uptake, as opposed to identifying as a Catholic or not following a religion. In addition to specific religious groups among the Protestant Christians (such as Orthodox reformed), the reluctance towards HPV vaccination might be extended amongst a broader group of Protestant Christians because of the link with a sexually transmitted infection. Participation in screening programs has not been found to be lower in Dutch regions with higher proportion of religious groups [25], highlighting again the possible complementarity between screening and vaccination.

Contradicting results regarding the influence of HPV knowledge and perceptions on vaccination uptake have been reported [28]. Consistent with a Dutch study on acceptance of HPV vaccination [29], we obtained no differences on vaccination uptake with regard to the general HPV knowledge score, nor on the HPV transmission knowledge score. Regardless of vaccination status, as many as 80% of the girls knew of the relationship between HPV and cervical cancer, which was similar to some other studies (81% Lenselink CH et al. [29], 84% Gerend MA and Shepherd JE [28], 85-93% Marlow LA et al. [30] program.

In general, only 50% of the young girls in our study recognised that after an HPV vaccination it is still indicated to participate in the cervical cancer screening program (which takes place from age 30 onwards in the Netherlands). This fact has also been reported by Bowyer HL et al. [31] amongst a group of 15–16 year old girls, where only 47% was aware cervical screening is still necessary after vaccination. We also found that vaccinated girls were less aware of the fact that vaccination does not protect against all HPV types. The continued importance of cervical screening in the Netherlands has to be emphasised along with the benefits of HPV vaccination. The finding that vaccinated girls report more often that their mothers participate in the cervical cancer screening program seems somewhat contradictory to their lower awareness about this program. Paulussen TG et al. [32] reported that a major part of the population does not make a well thought decision with regard to vaccinations in the regular National Immunization Program). These parents regard vaccination as self-evident. Similarly, this might be so for a part of mothers with regard to participation in screening, which possibly results in less discussion on the topic and thus less knowledge among their daughters.

In our study, only 2-3% reported that HPV vaccination had changed their sexual behaviour in the first year after vaccination. However, Marlow LA et al. [33] found that one-third of adolescent girls interviewed, thought that HPV vaccination would make girls in general more likely to have unsafe sex. Swedish high-school students did not think it that they themselves would engage in more unsafe sex after HPV vaccination but that other girls might [34]. We found a similar opinion concerning anticipated risk behaviour and condom use. Nearly all girls reported that they would not reduce condom use after HPV vaccination (only 1% for both groups), but 17% (vaccinated) and 26% (unvaccinated) thought other girls would.

There were no significant differences amongst vaccinated and unvaccinated girls for reported condom use with a casual or steady partner. However, sexually active vaccinated girls were more aware of the risk of HPV infection when engaging in unprotected sex. Irrespective of vaccination status, only a quarter of the girls reported to always use condoms with a casual partner and 18% with a steady partner. In line with Mather T et al. [35] inconsistent or no condom use was reported by 50% of the girls with a casual partner. Although most of the girls (65%) in our study reported using other contraceptive methods, the risk of acquiring HPV or another STI through unprotected sex seems to be high in this population. Therefore, early HPV vaccination of 12-year-old girls will considerably decrease the risk of HPV infection at a later and more sexually active age.

With regard to other sexual risk factors we found that among the vaccinated sexually active girls, a somewhat lower mean number of lifetime sexual partners was reported. However, no differences were found for other sexual behavioural characteristics such as age of sexual debut and history of STIs. These results imply that girls who decided not to get vaccinated were not the ones with increased (sexual) risk behaviour. Thus in contrast to screening, where it has been found that more than half of the cervical cancer cases were found in women who did not attend screening, it seems that although these girls are not benefiting from vaccination, they have no disproportional disadvantage compared to vaccinated girls.

This study has several strengths. Firstly, it was a randomly selected, large, nationwide population-based study. Secondly, vaccination status was derived from a national vaccination database instead of being self-reported. Thirdly, we obtained information on the actual vaccination uptake instead of the intention to vaccinate.

Although a limitation of this study was a relatively low response rate (15%), online surveys/questionnaires, while advantageous, are known for their average lower response rate compared to mail or telephone surveys [36,37]. Similar or lower response rates were obtained in other studies directed to parents of girls targeted for HPV catch-up vaccination (16%-24%) [38] or to participants recruited from Praeventis (7%) [39]. The response was higher amongst vaccinated than unvaccinated girls (19% vs. 11%) similar to other studies [38]. In order to assess the effect of this response rate we determined if the characteristics of the study population were similar to those of girls in the general population, by comparing our data on education, ethnicity and age of sexual debut. Girls in our study were comparable with respect to sexual debut with another Dutch study [40] but were slightly higher educated than the general Dutch population (59% vs. 47%), and were more often not Dutch (4.7% vs. 3.3%) [41]. Nevertheless, our sample size was adequate for the proposed statistical analysis of association with vaccine uptake and the possible influence of differences in ethnicity and education were controlled for in the multivariable analysis. Finally, although we have no indications that our results are not applicable to 12-year old girls that are targeted in the routine HPV vaccination program, we cannot rule out that this might be somewhat different. That is, the decision-process towards vaccine uptake may be more influenced by parents/caregivers in this younger age group.

## Conclusions

Routine HPV vaccination in the Netherlands has the potential to reduce the inequity of prevention of cervical cancer. In particular, vaccination uptake was not associated with factors that are known to be associated with non-attendance in the cervical cancer screening program, such as education and ethnicity. Furthermore, most sexual characteristics were comparable amongst both groups indicating that unvaccinated girls are probably not at higher risk of exposure to HPV compared to vaccinated girls. Although in general knowledge could be improved, the large majority of participants knew HPV caused cervical cancer suggesting an informed vaccination choice was made.

## Competing interest

Tjalke Westra (TAW) was involved in this study during his PhD at the University Medical Center Groningen, on an unrestricted educational grant from GlaxoSmithKline (GSK). Currently, TAW is employee of GSK. The other authors declare that they have no conflict of interest.

## Authors’ contributions

KL, MM, AT, HEDM were involved in the data collection, made substantial intellectual contributions to the conceptualization and design of this study and contributed to the content and preparation of this manuscript. MABVDS, SKS, TD, WCMWS, HWN, TAW made substantial intellectual contributions to the conceptualization of this study, the critical reading of the manuscript and final approval of the version to be published. All authors read and approved the final manuscript.

## Authors’ information

Madelief Mollers and Karin Lubbers shared first authorship.

## Pre-publication history

The pre-publication history for this paper can be accessed here:

http://www.biomedcentral.com/1471-2458/14/288/prepub

## References

[B1] Baseman JG Koutsky LA The epidemiology of human papillomavirus infections J Clin Virol 2005 32 Suppl 1 16 24 10.1016/j.jcv.2004.12.008 15753008

[B2] Koutsky L Epidemiology of genital human papilloma virus infection Am J Med 1997 102 Suppl 5A 3 8 9217656 10.1016/s0002-9343(97)00177-0

[B3] Brown DR Shew ML Qadadri B Neptune N Vargas M Tu W Juliar BE Breen TE Fortenberry JD A longitudinal study of genital human papillomavirus infection in a cohort of closely followed adolescent women J Infect Dis 2005 191 182 192 10.1086/426867 15609227 PMC2586143

[B4] Ho G Bierman R Beardsley L Chang CJ Burk RD Natural history of cervicovaginal papillomavirus infection in young women N Engl J Med 1998 338 423 428 10.1056/NEJM199802123380703 9459645

[B5] Moscicki A-B Schiffman M Kjaer S Villa LL Chapter 5: updating the natural history of HPV and anogenital cancer Vaccine 2006 24 Suppl 3 42 51 10.1016/j.vaccine.2006.06.018 16950017

[B6] zur Hausen H Gissmann L Steiner W Dippold W Dreger J Human papilloma viruses and cancer Bibl Haematol 1975 43 569 571 183728 10.1159/000399220

[B7] Castellsagué X Muñoz N Pitisuttithum P Ferris D Monsonego J Ault K Luna J Myers E Mallary S Bautista OM Bryan J Vuocolo S Haupt RM Saah A End-of-study safety, immunogenicity, and efficacy of quadrivalent HPV (types 6, 11, 16, 18) recombinant vaccine in adult women 24–45 years of age Br J Cancer 2011 105 28 37 10.1038/bjc.2011.185 21629249 PMC3137403

[B8] Lehtinen M Paavonen J Wheeler CM Jaisamrarn U Garland SM Castellsagué X Skinner SR Apter D Naud P Salmerón J Chow S-N Kitchener H Teixeira JC Hedrick J Limson G Szarewski A Romanowski B Aoki FY Schwarz TF Poppe WJ De Carvalho NS Germar MJV Peters K Mindel A De Sutter P Bosch FX David M-P Descamps D Struyf F Dubin G Overall efficacy of HPV-16/18 AS04-adjuvanted vaccine against grade 3 or greater cervical intraepithelial neoplasia: 4-year end-of-study analysis of the randomised, double-blind PATRICIA trial Lancet Oncol 2012 13 89 99 10.1016/S1470-2045(11)70286-8 22075171

[B9] van Lier EAOP Giesbers H Drijfhout IH de Hoogh PAAM de Melker HE Vaccinatiegraad Rijksvaccinatieprogramma Nederland: Verslagjaar 2012 RIVM rapport nr. 201001001 2012 Bilthoven: Rijksinstituut voor Volksgezondheid en Milieu

[B10] Brabin L Roberts SA Stretch R Baxter D Elton P Kitchener H McCann R A survey of adolescent experiences of human papillomavirus vaccination in the Manchester study Br J Cancer 2009 101 1502 1504 10.1038/sj.bjc.6605362 19809431 PMC2778524

[B11] Chao C Velicer C Slezak JM Jacobsen SJ Correlates for completion of 3-dose regimen of HPV vaccine in female members of a managed care organization Mayo Clin Proc 2009 84 864 870 10.4065/84.10.864 19797775 PMC2755805

[B12] Bulk S Visser O Rozendaal L Verheijen RH Meijer CJ Cervical cancer in the Netherlands 1989–1998: decrease of squamous cell carcinoma in older women, increase of adenocarcinoma in younger women Int J Cancer 2005 113 6 1005 1009 10.1002/ijc.20678 15515017

[B13] Rebolj M van Ballegooijen M Berkers LM Habbema D Monitoring a national cancer prevention program: successful changes in cervical cancer screening in the Netherlands Int J Cancer 2007 120 4 806 812 10.1002/ijc.22167 17131311

[B14] van Leeuwen AW de Nooijer P Hop WC Screening for cervical carcinoma Cancer 2005 105 270 276 10.1002/cncr.21153 15937918

[B15] van der Aa MA Schutter EM Looijen-Salamon M Martens JE Siesling S Differences in screening history, tumour characteristics and survival between women with screen-detected versus not screen-detected cervical cancer in the east of The Netherlands, 1992–2001 Eur J Obstet Gynecol Reprod Biol 2008 139 204 209 10.1016/j.ejogrb.2007.10.017 18093720

[B16] Bulk S Visser O Rozendaal L Verheijen RH Meijer CJ Incidence and survival rate of women with cervical cancer in the Greater Amsterdam area Br J Cancer 2003 89 834 839 10.1038/sj.bjc.6601157 12942114 PMC2394479

[B17] Dillard JP An application of the integrative model to women’s intention to be vaccinated against HPV: implications for message design Health Commun 2011 26 479 486 10.1080/10410236.2011.554170 21452094

[B18] Kang HS Moneyham L Attitudes toward and intention to receive the human papilloma virus (HPV) vaccination and intention to use condoms among female Korean college students Vaccine 2010 28 811 6 10.1016/j.vaccine.2009.10.052 19879993

[B19] Kim HW Knowledge about human papillomavirus (HPV), and health beliefs and intention to recommend HPV vaccination for girls and boys among Korean health teachers Vaccine 2012 30 5327 5334 10.1016/j.vaccine.2012.06.040 22749602

[B20] Tiro JA Tsui J Bauer HM Yamada E Kobrin S Breen N Human papillomavirus vaccine use among adolescent girls and young adult women: an analysis of the 2007 california health interview survey J Womens Health 2012 21 656 665 10.1089/jwh.2011.3284 PMC3412579 22420920

[B21] Kessels SJM Marshall HS Watson M Braunack-Mayer AJ Reuzel R Tooher RL Factors associated with HPV vaccine uptake in teenage girls: a systematic review Vaccine 2012 30 3546 3556 10.1016/j.vaccine.2012.03.063 22480928

[B22] van Lier A Oomen P de Hoogh P Drijfhout I Elsinghorst B Kemmeren J Conyn-van Spaendonck M de Melker H Praeventis, the immunisation register of the netherlands: a tool to evaluate the national immunisation program Euro Surveill 2012 17 17 10.2807/ese.17.17.20153-en 22551495

[B23] Helsinki declaration http://www.wma.net/en/30publications/10policies/b3

[B24] Knol F From high to low; from low to high: the social development of districts between 1971 and 1995 [in dutch]. The hague; the Netherlands institute for social research 1998

[B25] Steens A Wielders CC Bogaards JA Boshuizen HC de Greeff SC de Melker HE Association between human papillomavirus vaccine uptake and cervical cancer screening in the Netherlands: implications for future impact on prevention Int J Cancer 2013 132 932 943 10.1002/ijc.27671 22689326

[B26] Grabenstein JD What the world’s religions teach, applied to vaccines and immune globulins Vaccine 2013 31 16 2011 2023 10.1016/j.vaccine.2013.02.026 23499565

[B27] White A Theological opposition to inoculation, vaccination, and the use of anæsthetics. A history of the warfare of science with theology in Christendom. Chapter 13. SAGE software, albany, oregon 1996 New York: D. Appleton and Company Accessible at: http://vserver1.cscs.lsa.umich.edu/~crshalizi/White/

[B28] Gerend MA Shepherd JE Correlates of HPV knowledge in the era of HPV vaccination: a study of unvaccinated young adult women Women Health 2011 51 25 40 10.1080/03630242.2011.540744 21391159 PMC3080102

[B29] Lenselink CH Schmeink CE Melchers WJ Massuger LF Hendriks JC van Hamont D Bekkers RL Young adults and acceptance of the human papillomavirus vaccine Public Health 2008 122 1295 1301 10.1016/j.puhe.2008.02.010 18619631

[B30] Marlow LA Zimet GD McCaffery KJ Ostini R Waller J Knowledge of human papillomavirus (HPV) and HPV vaccination: an international comparison Vaccine 2012 31 763 769 23246310 10.1016/j.vaccine.2012.11.083

[B31] Bowyer HL Marlow LA Hibbitts S Pollock KG Waller J Knowledge and awareness of HPV and the HPV vaccine among young women in the first routinely vaccinated cohort in England Vaccine 2013 31 1051 1056 10.1016/j.vaccine.2012.12.038 23277094

[B32] Paulussen TG Hoekstra F Lanting CI Buijs GB Hirasing RA Determinants of Dutch parents’ decisions to vaccinate their child Vaccine 2006 24 5 644 651 10.1016/j.vaccine.2005.08.053 16157423

[B33] Marlow LAV Forster AS Wardle J Waller J Mothers’ and adolescents’ beliefs about risk compensation following HPV vaccination J Adolesc Health 2009 44 446 451 10.1016/j.jadohealth.2008.09.011 19380091

[B34] Höglund AT Tydén T Hannerfors AK Larsson M Knowledge of human papillomavirus and attitudes to vaccination among Swedish high school students Int J STD AIDS 2009 20 102 107 10.1258/ijsa.2008.008200 19182055

[B35] Mather T McCaffery K Juraskova I Does HPV vaccination affect women’s attitudes to cervical cancer screening and safe sexual behaviour? Vaccine 2012 30 3196 3201 10.1016/j.vaccine.2012.02.081 22425789

[B36] Monroe M Adams D Increasing response rates to web-based surveys Journal of Extension [Online] 2012 50 6 Article 6TOT7

[B37] Bergeson SC Gray J Ehrmantraut LA Laibson T Hays RD Comparing Web-based with mail survey administration of the consumer assessment of healthcare providers and systems (CAHPS) clinician and group survey Prim Health Care 2013 3 1000132 24078901 10.4172/2167-1079.1000132 PMC3783026

[B38] Gefenaite G Smit M Nijman HW Tami A Drijfhout IH Pascal A Postma MJ Wolters BA van Delden JJ Wilschut JC Hak E Comparatively low attendance during human papillomavirus catch-up vaccination among teenage girls in the Netherlands: insights from a behavioral survey among parents BMC Public Health 2012 12 498 10.1186/1471-2458-12-498 22748022 PMC3461412

[B39] van Keulen HM Otten W Ruiter RA Fekkes M van Steenbergen J Dusseldorp E Paulussen TW Determinants of HPV vaccination intentions among Dutch girls and their mothers: a cross-sectional study BMC Public Health 2013 13 111 10.1186/1471-2458-13-111 23388344 PMC3570492

[B40] de Graaf H Meijer S Poelman J Sex Under 25 [in Dutch] 2005 Delft, the Netherlands: Eburon Publishers, Rutgers Nisso Groep

[B41] Statistics Netherlands http://statline.cbs.nl/StatWeb

